# Translation into Portuguese of: "Changes to publication requirements made at the XVIII International Botanical Congress in Melbourne - what does e-publication mean for you?". Translated by Jefferson Prado, Regina Y. Hirai, and Cíntia Kameyama Alterações nos requisitos de publicação feitas no XVIII Congresso Internacional de Botânica em Melbourne – o que significa publicação eletrônica para você?

**DOI:** 10.3897/phytokeys.6.1985

**Published:** 2011-09-14

**Authors:** Sandra Knapp, John McNeill, Nicholas J. Turland

**Affiliations:** 1 Department of Botany, The Natural History Museum, Cromwell Road, London SW7 5BD, UK The Natural History Museum London United Kingdom; 2 Royal Botanic Garden, Edinburgh, 20A Inverleith Row, Edinburgh EH3 5LR, UK Royal Botanic Garden Edinburgh United Kingdom; 3 Missouri Botanical Garden, PO Box 299, St Louis, MO 63166-0299, USA Missouri Botanical Garden St. Louis United States of America

## Abstract

Alterações no *Código Internacional de Nomenclatura Botânica* são decididas a cada seis anos na Sessão de Nomenclatura, associada ao Congresso Internacional de Botânica (CIB). O XVIII CIB foi realizado em Melbourne, Austrália; a Sessão de Nomenclatura ocorreu de 18-22 de julho de 2011 e suas decisões foram aceitas pelo Congresso, em sua sessão plenária em 30 de julho. Como resultado desta reunião, foram decididas várias mudanças importantes no *Código* que afetarão a publicação de novos nomes. Duas dessas mudanças se tornarão efetivas em 1° de janeiro de 2012, alguns meses antes do *Código de Melbourne* ser publicado. Material eletrônico publicado online em Formato de Documento Portátil (PDF) com um Número Padrão Internacional de Séries (ISSN) ou com um Número Padrão Internacional de Livros (ISBN) constituirão publicação efetiva e o requisito de uma descrição ou diagnose em latim para nomes de táxons novos será substituído por uma descrição ou diagnose em latim ou inglês. Além disso, nomes novos de organismos tratados como fungos devem, a partir de 1° de janeiro de 2013, a fim de ser validamente publicados, incluir no seu protólogo a citação de um número identificador de um indexador de nomes (p.e., MycoBank). O texto preliminar de novos artigos, tratando de publicação eletrônica, é apresentado e o melhor modo de fazer isto é discutido.

Para encorajar a disseminação das mudanças feitas no *Código Internacional de Nomenclatura para algas, fungos e plantas*, este artigo será publicado na *BMC Evolutionary Biology, Botanical Journal of the Linnean Society, Brittonia, Cladistics, MycoKeys, Mycotaxon, New Phytologist, North American Fungi, Novon, Opuscula Philolichenum, PhytoKeys, Phytoneuron, Phytotaxa, Plant Diversity and Resources, Systematic Botany* e *Taxon*.

## Introdução

No XVIII Congresso Internacional de Botânica em Melbourne, Austrália, em julho de 2011, duas alterações importantes foram decididas para o *Código Internacional de Nomenclatura Botânica* (agora, o *Código Internacional de Nomenclatura para algas, fungos e plantas*) e se tornarão efetivas a partir de 1° de janeiro de 2012. Essas mudanças afetarão todos aqueles que publicam nomes regidos por este *Código*. Como o *Código de Melbourne* não será publicado até aproximadamente a metade de 2012, nós achamos que seria útil esboçar essas mudanças, particularmente aquelas relacionadas à publicação efetiva em mídia eletrônica (nos Artigos 29, 30 e 31). Um relatório conciso de todas as alterações para o *Código* aceitas em Melbourne, pode ser visto em McNeill et al. (2011).

Um rascunho revisado da redação dos Artigos, Notas e Recomendações sobre publicação efetiva é apresentado para auxiliar os editores e editoras no estabelecimento de boas práticas para implementação deste aspecto do *Código*. Nós também esboçamos aqui o que essas mudanças *não* significam, para guiar aqueles que desejam publicar nomes novos e tipificações por meios eletrônicos. Nós recomendamos aos leitores consultar o relatório do Comitê Especial sobre Publicação Eletrônica que acompanha as mudanças propostas anteriormente ao Congresso (Chapman et al. 2010), onde estão as razões para estas alterações agora aceitas no Código e aqui expostas.

## Rascunho revisado da redação dos Artigos 29, 30 e 31 e Recomendações 29A, 30A e 31A

Nós reproduzimos aqui a redação de todos os Artigos, Notas e Recomendações relevantes (omitindo os Exemplos), com as mudanças destacadas em **negrito**. A redação aqui é provisória, dependendo da reunião do Comitê Editorial em dezembro de 2011 para finalizar a versão impressa do *Código de Melbourne*.

### Artigo 29

*29.1*. A publicação é efetiva, segundo este *Código*, somente pela distribuição de matéria impressa (por meio de venda, intercâmbio ou doação) ao público em geral ou, pelo menos, às instituições botânicas com bibliotecas acessíveis aos botânicos em geral. **A publicação também é efetiva pela distribuição eletrônica da matéria em Formato de Documento Portátil (PDF; veja também Art. 29.3 e Rec. 29A.1) em uma publicação online com um Número Padrão Internacional de Séries (ISSN) ou com um Número Padrão Internacional de Livros (ISBN).** Não é efetiva pela comunicação de nomes novos em reuniões públicas, pela colocação de nomes em coleções ou jardins abertos ao público, pela produção de microfilme feito a partir de manuscritos, textos datilografados ou outro material não publicado, ou pela distribuição eletrônica ou por qualquer meio eletrônico, **exceto como descrito acima**.

***29.2.* Para propósito deste Artigo, “online” é definido como acessível eletronicamente via “World Wide Web”.**

***29.3.* Caso o Formato de Documento Portátil (PDF) seja sucedido por um formato padrão internacional, este formato sucessor informado pelo Comitê Geral (veja Div. III), é aceitável.**

***29.4.* O conteúdo de uma publicação eletrônica em particular não deve ser alterado depois dele publicado pela primeira vez. Nenhuma dessas alterações é efetivamente publicada. Correções ou revisões devem ser publicadas separadamente para serem efetivamente publicadas.**

### Recomendação 29A

[A Recomendação atual será substituída pelas seguintes]

***29A.1.* A publicação eletrônica em Formato de Documento Portátil (PDF) deveria estar de acordo com o padrão de arquivo PDF/A (ISO 19005).**

***29A.2.* Autores deveriam preferencialmente publicar em publicações que são arquivadas, cumprindo os seguintes critérios tanto quanto possível na prática (veja também Rec. 29A.1):**

***(a)* O material deveria ser colocado em múltiplos repositórios digitais online confiáveis, p.e., um repositório com certificado ISO;**

***(b)* Repositórios digitais deveriam estar em mais de um local do mundo e preferencialmente em diferentes continentes;**

***(c)* É também recomendável o depósito de cópias impressas em bibliotecas em mais de um local do mundo e preferencialmente em diferentes continentes.**

### Article 30

***30.1.* Publicação através da distribuição de matéria eletrônica não constitui publicação efetiva antes de 1° de janeiro de 2012.**

***30.2.* Uma publicação eletrônica não é efetivamente publicada caso exista uma evidência associada com ou dentro dela de que ela seja meramente uma versão preliminar, que foi ou que será substituída por uma versão que o editor considera final, neste caso somente a versão final é efetivamente publicada.**

*30.3*. Publicação através de manuscrito indelével é efetiva antes de 1° de janeiro de 1953. Manuscrito indelével produzido em data posterior não é efetivamente publicado.

*30.4.* Para fins deste artigo, manuscrito indelével é o material manuscrito reproduzido por algum processo mecânico ou gráfico (tal como litografia, ‘offset’ ou gravação em chapa metálica).

*30.5.* Publicação em ou a partir de 1° de janeiro de 1953, em catálogos de intercâmbio ou em revistas não-científicas e em ou a partir de 1° de janeiro de 1973, em listas de intercâmbio de sementes, não constitui publicação efetiva.

*30.6.* A distribuição em ou a partir de 1° de janeiro de 1953, de material impressa que acompanhe exsicatas não constitui publicação efetiva.

*Nota 1.* Se a matéria impressa também for distribuída independente da exsicata, constitui publicação efetiva.

*30.7.* Publicação em ou a partir de 1° de janeiro de 1953, de um trabalho independente, não seriado, definido como uma tese submetida a uma Universidade ou outro instituto de educação com o propósito de obtenção de grau, não é efetivamente publicada, a menos que inclua uma declaração explícita (referente aos requisitos do *Código* para publicação efetiva) ou outra evidência interna de que seja entendida como uma publicação efetiva por seu autor ou editor.

*Nota 2.* A presença de um Número Padrão Internacional de Livros (ISBN) ou a colocação do nome da gráfica, da editora ou distribuidor na versão impressa original é entendido como evidência interna de que o trabalho foi definido como efetivamente publicado.

### Recommendation 30A

30A.1. **Versões preliminares e finais da mesma publicação eletrônica deveriam ser claramente indicadas tal como quando são publicadas pela primeira vez.**

*30A.2.* É fortemente recomendado que os autores evitem publicar nomes novos e descrições ou diagnoses de táxons novos (novidades nomenclaturais) em matéria impressa de qualquer tipo que seja efêmera, em matéria impressa particular que seja multiplicada em números restritos e incertos, nos quais a permanência do texto possa ser limitada, para os quais a publicação efetiva em termos de número de cópias não seja óbvia ou que seja improvável que alcance o público em geral. Os autores deveriam também evitar nomes novos e descrições ou diagnoses em revistas populares, em revistas de resumos (‘abstracting journals’) ou em etiquetas de correção.

*30A.3.* Para ajudar na disponibilidade em termos de tempo e lugar, os autores ao publicar novidades nomenclaturais, deveriam dar preferência a revistas periódicas que regularmente publiquem artigos taxonômicos. **Por outro lado,** **uma cópia da publicação (se publicada como matéria eletrônica ou impressa) deveria ser enviada para um centro indexador apropriado do grupo taxonômico e publicações que existirem somente como matéria impressa** deveriam ser depositadas em, pelo menos dez, mas preferivelmente em mais bibliotecas botânicas ou outras geralmente acessíveis em todo o mundo.

*30A.4.* Autores e editores são encorajados a mencionar as novidades nomenclaturais no resumo ou no ‘abstract’ ou listá-las num índice na própria publicação.

### Article 31

*31.1.* A data de publicação efetiva é a data na qual a matéria impressa **ou eletrônica** tornou-se disponível conforme definido nos Art. 29 e 30. Na ausência de prova estabelecendo alguma outra data, a data que aparece na matéria impressa **ou eletrônica** deve ser aceita como correta.

[A Nota 1 atual será substituída pelo seguinte]

***31.2.* Quando uma publicação é lançada paralelamente em versões eletrônica e impressa, estas devem ser tratadas como efetivamente publicadas na mesma data a menos que as datas das versões sejam diferentes de acordo com o Art. 31.1.**

*31.3.* Quando separatas de periódicos ou outros trabalhos colocados à venda são distribuídos antes, a data na separata é aceita como a data da publicação efetiva, a menos que haja evidência de que esteja errada.

### Recomendação 31A

*31A.1.* A data na qual o editor ou o agente do publicador distribuiu a matéria impressa a um dos transportadores usuais para distribuição ao público deveria ser aceita como a data de publicação efetiva.

## Boas práticas

Autores de nomes novos, editores e editoras estarão todos interessados em assegurar que as publicações incluindo nomes novos estejam de acordo com o *Código de Melbourne*, de tal modo que os nomes sejam efetivamente publicados. Nós sugerimos que aqueles que publicam, em periódicos ou monografias seriadas e livros que tenham edições online, se comuniquem com os editores de modo que as boas práticas possam ser estabelecidas na comunidade, o mais rápido possível. Já há algum tempo, muitos editores tem sido cuidadosamente orientados sobre as novidades relacionadas às publicações eletrônicas (veja Knapp and Wright 2010; guia para autores em PLoS One [http://www.plosone.org/static/policies.action#taxon] e eles tem, aparentemente, um considerável interesse em tornar essas novas mudanças do *Código* efetivas.

Nós acreditamos que algumas práticas que ajudarão nos estágios iniciais das publicações eletrônicas de novidades, que estão de acordo com o *Código de Melbourne*, são:

• Cada artigo ter a data de publicação bem evidente (como é feito em vários periódicos, p.e., *New Phytologist* ou *Nature*).
• Se uma versão anterior online é publicada e esta não for igual à versão final (e deste modo não é o local de publicação efetiva), estampar em cada artigo esta informação de forma bem evidente (p.e., *American Journal of Botany*).
• A exposição bem evidente do ISSN ou ISBN da publicação em cada artigo ajudará os indexadores estabelecer a publicação efetiva.
• A publicação em periódicos (ou monografias seriadas) que participam do sistema CLOCKSS (veja Knapp and Wright 2010 para uma descrição) ou de outro sistema internacional de arquivo e preservação garantirá o arquivamento a longo prazo.
• Autores de nomes novos por meio eletrônico deveriam alertar o centro indexador apropriado como recomendado na Rec. 30A.3 – isto ajudará os indexadores que poderiam não estar cientes dos nomes publicados eletronicamente.

## O que essas mudanças *não* significam

Embora os novos Artigos e Recomendações usem os termos PDF e PDF/A, isto não significa que as publicações devam ser lançadas *somente* neste formato para ser efetivamente publicadas. Por exemplo, alguns periódicos online publicam trabalhos em formato HTML (Linguagem de Marcação de Hipertexto) junto com uma versão paralela em PDF. Nestes casos, a versão PDF será a efetivamente publicada. A determinação de que o Comitê Geral para Nomenclatura Botânica informará a aceitação de um novo formato padrão internacional, que poderá suceder o PDF, significa que autores de novidades e a comunidade que usam o *Código* permanecerão informados sobre os avanços na área e que o *Código* estará protegido da obsolescência.

O uso dos seguintes meios de publicação eletrônica *não* resultará em publicação efetiva de novidades sob o *Código* de Melbourne:

• A publicação em sítios na Web ou em documentos efêmeros disponíveis na Internet (há critérios estritos para concessão de ISSNs [http://www.issn.org]).
• A publicação em periódicos sem um ISSN ou e-ISSN registrado.
• A publicação em livros sem um ISBN ou e-ISBN registrado.

A Recomendação aprovada para orientar o depósito de uma cópia impressa de qualquer publicação eletrônica em uma biblioteca sugere aos botânicos uma ação, mas isto não estabelece uma prática padrão ou um protocolo para os bibliotecários seguirem. Os bibliotecários encontram-se em uma zona de transição complexa entre modalidades de publicação (Johnson and Luther 2007) e os botânicos podem encontrá-los não dispostos ou capacitados para acomodar cópias impressas avulsas de trabalhos como entradas pontuais, se o volume for grande.

## Outras duas mudanças importantes para o *Código* relacionadas à publicação de nomes

A segunda mudança para o *Código*, aprovada em Melbourne, que entrará em vigor a partir de 1° de janeiro de 2012 é que a descrição ou diagnose, requerida para publicação válida de um nome de um novo táxon de todos os organismos, sob este *Código*, pode ser em inglês ou em latim. Esta é uma provisão corrente para nomes de plantas fósseis, porém todos os táxons novos não-fósseis requeriam uma diagnose ou descrição em latim (fungos e plantas desde 1° de janeiro de 1935; algas [incluindo cianobactérias, se tratadas sob este *Código*] desde 1° de janeiro de 1958). Isto não se refere à forma de nomes científicos, que continuam sendo em latim ou tratados em latim. Os requisitos de cada periódico para latim e/ou inglês, deverão, claro, ser determinados pelos editores desses periódicos.

A terceira mudança para o *Código* aprovada em Melbourne está relacionada à publicação de nomes, porém não terá efeito até 1° de janeiro de 2013 (não em 1° de janeiro de 2012 como relatado por Miller et al. 2011), é que todos os nomes novos de organismos tratados como fungos devem, como um requisito adicional para publicação válida, incluir no protólogo (tudo associado com o nome na sua publicação válida) a citação de um número identificador de um repositório reconhecido (p.e., MycoBank [http://www.mycobank.org/]). Isto será divulgado à parte.

O requisito para um único identificador para nomes novos de fungos em ou a partir de 1° de janeiro de 2013 *não* se aplica a plantas ou algas; não há necessidade que autores de nomes novos nesses grupos requeram “Life Science Identifiers” (LSIDs) – ou outros identificadores – de centros indexadores.
